# Improvement of health-related quality of life in depression after transcranial magnetic stimulation in a naturalistic trial is associated with decreased perfusion in precuneus

**DOI:** 10.1186/1477-7525-10-87

**Published:** 2012-07-28

**Authors:** Rémy Dumas, Raphaëlle Richieri, Eric Guedj, Pascal Auquier, Christophe Lancon, Laurent Boyer

**Affiliations:** 1 Department of Psychiatry, Sainte-Marguerite University Hospital, Marseille, 13009, France; 2 Aix-Marseille Univ, EA 3279 - Self-perceived Health Assessment Research Unit, Marseille, 13005, France; 3 Service Central de Biophysique et Médecine Nucléaire, La Timone University Hospital, Assistance Publique - Hôpitaux de Marseille, Marseille, 13005, France; 4 Aix-Marseille Univ, CERIMED, Marseille, 13005, France; 5 Aix-Marseille Univ, INT, CNRS UMR 7289, Marseille, 13005, France; 6 Department of Public Health, Assistance Publique - Hôpitaux de Marseille, Marseille, France

**Keywords:** Depression, Health-related quality of life, Repetitive Transcranial Magnetic Stimulation (rTMS), Single photon emission computed tomography (SPECT), Precuneus

## Abstract

**Background:**

Assessing Health-related Quality of life (HRQoL) is necessary to evaluate care and treatments provided to patients with major depressive disorder (MDD), in addition to the traditional assessment of clinical outcomes. However, HRQoL remains under-utilized to assess the effectiveness of repetitive transcranial magnetic stimulation (rTMS) in research or in a routine clinical setting. The primary objective of this exploratory study on MDD was to investigate the impact of low-frequency rTMS on HRQoL using the SF-36 questionnaire. A secondary objective was to study the functional neural substrate underlying HRQoL changes using neuroimaging.

**Methods:**

Fifteen right-handed patients who met DSM-IV criteria for MDD participated in the study. HRQoL was assessed using the SF-36, and regional cerebral blood (rCBF) flow using 99mTc-ECD-SPECT. Voxel based correlation was searched between concomitant changes in rCBF and in HRQoL after rTMS.

**Results:**

Role-Physical Problems dimension showed a statistical significant improvement of 73.2% (p = 0.001) and an effect size (Cohen’s d) of 0.43, indicating moderate effect. Five SF-36 dimension scores and the two composite scores showed effect sizes ranged from 0.28 to 0.43. Improvement of Mental Composite Score (MCS)-SF-36 after rTMS was correlated with a concomitant decrease of precuneus perfusion (p < 0.001). Post-hoc analyses confirmed that decreased perfusion in precuneus was correlated with improvement of HRQoL, especially for MCS (r = −0.71; p < 0.001), Mental Health (r = −0.81; p < 0.001) and Social Functioning (r = −0.57; p = 0.026) dimensions.

**Conclusions:**

This study suggests low-frequency rTMS can improve HRQoL, through its role-physical problems dimension, in patients with MDD. This improvement is associated with a decreased perfusion of the precuneus, a brain area involved in self-focus and self-processing, arguing for a neural substrate to the impact of rTMS on HRQoL.

## Background

Major depressive disorder (MDD) [1] remains one of the leading causes of disability in developed countries despite pharmacological and psychological treatments [2]. A new alternative for depressed individuals is repetitive transcranial magnetic stimulation (rTMS), a non-invasive well-tolerated technique [3-5]. rTMS protocols have been developed using high-frequency stimulation (> 5 Hz) of the left dorsolateral prefrontal cortex (DLPFC) or low-frequency (< 1 Hz) of the right DLPFC. The choice of distinct frequency comes from electrophysiological data of the motor cortex, showing that high-frequencies may have an excitatory effect while low-frequencies tend to suppress cortical excitability [6,7]. Effectiveness of rTMS has been investigated in most studies using clinical outcomes, such as change in depressive symptomatology [8,9]. Thereby, meta-analyses have highlighted a threshold of 50% decrease in symptom severity, in up to 76% of patients [10-12]. Interestingly, the efficiency of low-frequency rTMS stimulation of the right DLPFC and of high-frequency rTMS stimulation of the left DLPFC seems to be similar in depressed patients [11,13,14].

On the other hand, Health-related Quality of life (HRQoL) questionnaires are considered valuable and necessary tools to evaluate care and treatments provided to patients with MDD, in addition to the traditional assessment of clinical outcomes [15-17]. HRQoL may add interesting information that is oriented toward a more global service to individuals including the subjective experience of their illness, with regard to their symptoms but also their functioning embedded in social and environmental context [15]. However, HRQoL remains under-utilized to assess the effectiveness of rTMS in research or in a routine clinical setting [8], and more globally in psychiatric clinical practice [18,19]. In congruence with prior investigations on HRQoL in MDD following antidepressant pharmacotherapy [20,21], only two recent studies, using the World Health Organization's Quality of Life Measure , brief version (WHOQOL BREF) [22] and the Medical Outcome Study 36-item Short Form (SF-36) [23,24] questionnaires, have shown improvement in HRQoL domains following high-frequency rTMS [8,25]. These important findings should be confirmed using low-frequency rTMS which is better tolerated than high-frequency rTMS in terms of seizure risk [26].

The primary objective of this exploratory study was to investigate the impact of low-frequency rTMS on HRQoL using the SF-36 questionnaire, in patients with MDD. In addition, a better understanding of the mechanism underlying HRQoL changes is of particular interest in depression, as already proposed in schizophrenia [27,28]. A secondary objective was thus to study the functional neural substrate underlying HRQoL changes using neuroimaging with 99 m Tc-ethyl cysteinate dimer single-photon emission computed tomography (99mTc-ECD-SPECT), a valuable tool to investigate the regional cerebral blood flow (rCBF), in a range of psychiatric disorders such as depression [29-31].

## Methods

### Patients

Fifteen right-handed patients, who met DSM-IV criteria for MDD participated in the study [1]. Inclusion criteria were: unipolar depression, Beck Depression Inventory-II (BDI-II) score ≥ 20 [32], non-response to pharmacological treatment using a minimum of two distinctly different classes of antidepressant medications for episodes occurring at the time of enrolment, and written informed consent. Exclusion criteria were based on the following criteria: age under 18 years, MDD with psychotic features, psychiatric diagnosis other than MDD on Axis I of DSM-IV-TR [1]*,* neurological disorders or convulsive disorders, and previous rTMS or electroconvulsive therapy treatments. Pretreatment with an antidepressant and/or mood stabilizer medication had to be stable for at least 4 weeks prior to entry in the study and remain unchanged throughout. The local Ethics Committee, Marseille, France approved the investigations. The project was conducted in accordance with the declaration of Helsinki and French Good Clinical Practice [33,34].

### rTMS treatment

Low-frequency magnetic stimulation was performed using a figure eight-shaped water-cooled coil (Medtronic Inc., Minneapolis, MN, USA) to the right DLPFC. At the first rTMS session, the motor threshold was defined as the minimum intensity leading to the most prominent abduction of the left abductor pollicis brevis muscle after stimulation of the right motor cortex. This movement was determined by an electromyogram recording. During the treatment, the coil was positioned 5 cm anterior and in a parasagittal line from the motor cortex. rTMS was delivered with following characteristics: six 60-s trains at 1 Hz and at 120% of the motor threshold were applied in each session with a 30-s interval between the trains. Twenty treatment sessions were administered during 4 weeks (total pulses, 7200) [11].

### Data collection

The following data were recorded: (1) demographic characteristics: gender and age, marital status and education level; (2) handedness determined by the Edinburgh Inventory [35]; (3) clinical characteristics: duration of illness, episode duration; (4) depression severity was assessed using the 21-item self-report Beck Depression Inventory (BDI-II) [32]. A score from 0 to 63 is calculated, with higher scores indicating more severe depressive symptoms; (5) HRQoL was assessed using the SF-36 [23,24]. SF-36 is a generic, self-administered, and worldwide-used questionnaire consisting of 36 items describing 8 dimensions: Physical Functioning (PF), Social Functioning (SF), Role-Physical Problems (RPP), Role-Emotional Problems (REP), Mental Health (MH), Vitality (VIT), Bodily Pain (BP), and General Health (GH). Each dimension is scored within a range of 0 (low HRQoL level) to 100 (high HRQoL level). Two component summary measures of SF-36, namely physical and mental composite scores (PCS-SF-36 and MCS-SF-36) are calculated.

Depression severity and HRQoL were assessed at baseline (t0), and at the end of rTMS (t1).

### SPECT protocol and analysis

Brain SPECT was performed in all patients, with the same camera, and under the same conditions, as previously described [29]. This exam was performed during the week before rTMS, and a second SPECT scan was obtained during the week after the end of the rTMS treatment. A voxel-by-voxel group study was then performed using Statistical Parametric Mapping (SPM) 8 (Wellcome Department of Cognitive Neurology, University College, London), running on Matlab (MathworksInc, Sherborn, MA).

Images were initially converted from the DICOM to the Analyze format using MRIcro (http://www.mricro.com), and transferred to SPM8. Data were then standardized with the Montreal Neurological Institute atlas, using a 12-parameter affine transformation, followed by non-linear transformations and trilinear interpolation. Dimensions of resulting voxels were 2x2x2 mm. Standardized data were then smoothed with a Gaussian filter (FWHM = 12 mm), to blur individual variations in gyral anatomy, and to increase signal-to-noise ratio. The “proportional scaling” routine was used to control for individual variation in global brain perfusion.

Voxel based correlation was searched between concomitant changes in rCBF and in HRQoL after rTMS (PCS- and MCS-SF-36 changes). For this, brain SPECT, PCS and MCS baseline scores were subtracted with their respective post rTMS scores, and then divided by their respective baseline scores. The SPM (T) maps were obtained at a height threshold of p < 0.001 (uncorrected) for the voxel, with at least 24 voxels within each cluster (forth FWHM of the Gaussian filter).

### Statistical analysis

Data were expressed in proportion or mean and standard deviation (SD). Pre-post rTMS HRQoL comparisons were analyzed using two-tailed paired t-tests. Cohen's d effect sizes were then calculated [8,36]. As recommended, we considered d values from 0.15 to 0.39, 0.4 to 0.74 and from 0.74 to 1.1 as indicating small, moderate and large effect sizes, respectively [8,36]. Associations were then looked for between cluster(s) previously found correlated with PCS- and/or MCS-SF-36, and changes in dimensions of SF-36 and BDI-II, using Spearman’s correlation tests. Spearman’s correlation tests were used to determine the relationship between changes in BDI-II score and changes in dimensions and scores of SF-36. All the tests were two-sided. Statistical significance was defined at p < 0.05. Statistical analysis was performed using the SPSS version 17.0 software package (SPSS Inc, Chicago, IL).

## Results

### Patient characteristics

The fifteen subjects (twelve women) presented pharmacoresistant unipolar depression with high severity (BDI-II = 36; SD = 9.8). The average patient age was 49.8 years (SD = 10.5), the duration of illness was 14.4 years (SD = 10.1), and the duration of current depressive episode was 38.2 months (SD = 35.1). Twelve patients were single or divorced; ten patients had a high school or college education. Most patients (n = 13) received rTMS as add-on treatment, and were treated under antidepressant monotherapy (n = 10), or combination of antidepressant and mood stabilizer (n = 3). Nine patients took benzodiazepines or hypnotics during rTMS treatment. Two patients were totally medication-free.

### Quality of life

PCS showed improvement of 5.1%, MCS of 13.9%, and BDI-II of 13.1%. Role-Physical Problems dimension showed a statistically significant improvement of 73.2% (p = 0.001) and an effect size (Cohen’s d) of 0.43, indicating moderate effect. General Health dimension remained fairly constant (2% increase), although the other dimensions increased from 11 (Social functioning) to 100% (Role-Emotional Problems). Five SF-36 dimension scores (Physical Functioning, Role-Emotional Problems, Mental Health, Bodily Pain, Vitality) and the two composite scores showed small to moderate effect sizes (Table 1 ). Significant correlations between BDI-II changes and HRQoL were found for the following dimensions: MCS (r = −0.71; p = 0.003), MH (r = −0.65; p = 0.009), SF (r = −0.58; p = 0.023), VIT (r = −0.75; p = 0.001), PF (r = −0.56; p = 0.03) and GH (r = −0.52; p = 0.04).

**Table 1:** Pre-post rTMS HRQoL comparisons (n = 15)

**SF-36**	**Pre-rTMS**	**Post-rTMS**	***P value***	**Effect size (ES)**
	**Mean (SD)**	**Mean (SD)**		**(Cohen's d)**
PF	53.3 (25.4)	62.0 (28.5)	0.193	+ 0.32 ^a^
RPP	18.3 (30.6)	31.7 (32.0)	0.001	+ 0.43 ^b^
VIT	19.3 (15.1)	25.0 (17.1)	0.270	+ 0.35 ^a^
BP	40.9 (21.2)	49.9 (28.7)	0.125	+ 0.36 ^a^
MH	25.6 (12.0)	32.8 (22.8)	0.247	+ 0.39 ^a^
REP	13.3 (24.6)	26.7 (36.1)	0.159	+ 0.43 ^b^
SF	29.2 (21.0)	32.5 (25.4)	0.638	+ 0.14
GH	28.6 (21.1)	29.1 (19.5)	0.937	+ 0.02
MCS	23.7 (6.8)	27 (12)	0.109	+ 0.31 ^a^
PCS	38.9 (8.1)	40.9 (9.2)	0.177	+ 0.28 ^a^

SF-36—*PF* Physical Functioning, *RPP* Role—Physical Problems, *VIT* Vitality, *BP* Bodily Pain, *MH* Mental Health, *REP* Role—Emotional Problems, *SF* Social Functioning, *GH* General Health, *MCS* Mental Composite Score, *PCS* Physical Composite Score.

^a^ small effect size; ^b^ moderate effect size.

### Correlation between brain SPECT perfusion and HRQOL and clinical scores after rTMS

**Figure 1:**
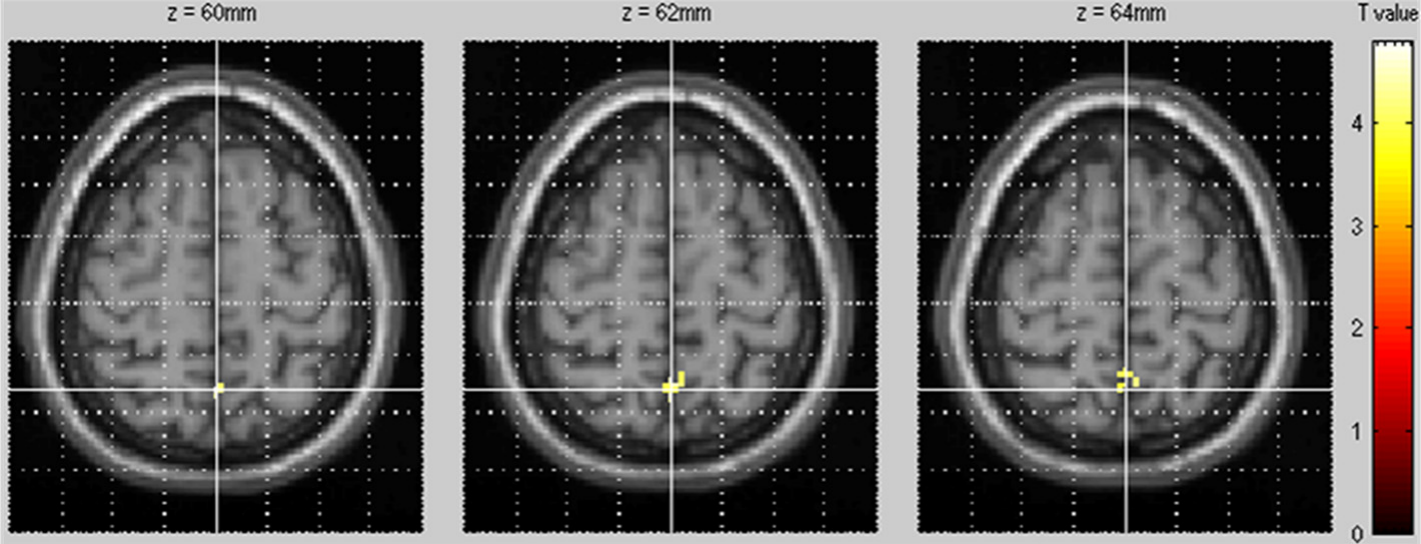
Anatomical localization of areas of decreased perfusion after rTMS correlated with concomitant HRQoL improvement (p < 0.001, uncorrected; k > 24).

HRQoL improvement for MCS-SF-36 was associated with a decreased perfusion of the precuneus (p < 0.001, uncorrected; T-score = 3.85) (Figure 1). No other significant correlation was found using whole brain analysis, in particular for PCS-SF-36 and for BDI-II.

Spearman’s correlations were then looked for between this cluster and the SF-36 dimension and BDI-II scores (Table 2). rCBF decrease of the precuneus was highly correlated with an HRQoL dimension increase of MCS-SF-36 (r = −0.71; p < 0.001), MH (r = −0.81; p < 0.001) and SF (r = −0.57; p = 0.026). On the contrary, precuneus perfusion was not significantly correlated with other SF-36 dimensions or with BDI-II score (p > 0.05).

**Table 2:** Correlations between changes in rCBF within the precuneus and changes in SF-36 dimensions and BDI-II

	**Precuneus cluster R**^**#**^	***p values***
**SF-36**		
PF	- 0.25	0.380
RPP	0.04	0.881
VIT	- 0.10	0.712
BP	0.23	0.410
MH	- 0.81	**< 0.001**
REP	- 0.37	0.179
SF	- 0.57	**0.026**
GH	- 0.10	0.712
MCS	- 0.71	**< 0.001**
PCS	0.37	0.173
**BDI-II**	0.46	0.087

# R: Spearman’s correlation coefficient.

SF-36—*PF *Physical Functioning, *RPP* Role—Physical Problems, *VIT *Vitality, *BP *Bodily Pain, *MH *Mental Health, *REP *Role—Emotional Problems, *SF *Social Functioning, *GH *General Health, *MCS *Mental Composite Score, *PCS *Physical Composite Score.

*BDI-II.* Beck Depression Inventory - II.

## Discussion

Our study provides important information on the impact of rTMS on HRQoL in patients with MDD. Firstly, using a low-frequency rTMS administered on the right DLPFC, our findings tend to confirm the preliminary results of Berlim et al.[8], and Hadley et al.[25] using a high-frequency rTMS on the left DLPFC. Role-Physical Problems dimension improved significantly from baseline and showed moderate effect size. Five SF-36 dimension scores and the two composite scores showed effect sizes higher than 0.28, suggesting a small to moderate “clinically” meaningful improvement of HRQoL. Although the HRQoL increase in our study showed less magnitude on physical, psychological and overall domains (respective Cohen’s d, 0.44, 0.44, 0.57) than those found in Berlim’s study [8] and on physical functioning, vitality, social functioning and mental health dimensions than in Hadley’s study [25], precaution should be taken in this comparison. This difference may be explained by a higher effect of high-frequency rTMS on HRQoL than low-frequency rTMS. However, the study of Berlim et al. did not use the same questionnaire, and the SF-36 and WHOQOL-BREF appear to measure distinct concepts related to HRQoL [37]. Concerning the study of Hadley et al., the baseline HRQoL levels were significantly higher than in our sample, and differential change in means from baseline may simply be due to skewness interacting with baseline differences [38]. Future studies should thus compare the impact of high and low-frequency rTMS on HRQoL using both questionnaires.

Moreover, this whole-brain voxel-based study is the first to investigate the neural substrate underlying HRQoL changes in patients with MDD treated by rTMS. We show that improvement of HRQoL for social and mental health dimensions SF-36-scores after rTMS is associated with a decreased perfusion of the precuneus. The precuneus has reciprocal connections with the DLPFC [39]. So, the decreased activity of the precuneus can result from the inhibitory effects of low-frequency rTMS over the right DLPFC, which can induce activity changes in distant brain regions via the neural pathways [40].

This report strengthens the scientific conceptual basis of HRQoL. The psychosocial construct of HRQoL, which required accurate self-assessment by individuals of their own inner feelings and state of well-being, appears consistent with precuneus involvement [31]. Indeed, precuneus has been mainly implicated in subjective experience and conscious self-representation [31,41]. In healthy subjects, precuneus is activated in self-related mental representations: at rest, through the default mode network [39], and during tasks about reflection on one’s own personality traits and physical appearance [42]. Precuneus has been also involved in the subjective well-being and distress of patients with post-traumatic stress disorder [43].

Our findings attempt to provide some clues for a better understanding of the mechanism underlying the relationship between depression and HRQoL found in previous studies [15,44,45]. Indeed, patients with depression suffer from an increased self-focus (i.e. link negative affect and episodic memory deficit with an increased attention to the self) [46], and exaggerated self-processing (i.e. the appraisal of stimuli as strongly related to one’s own person) [47]. Exaggerated self-referential processing (SRP) especially distorts interpretations of social cues and maintains social fears because of maladaptive cognitions regarding self (i.e. as socially incompetent) and others (i.e. as critical judges) [48]. Decreased activation in the precuneus could thus reflect a restored deactivation of the default mode network [49]. We hypothesize that decreased rCBF of the precuneus may be associated to a reduction of SRP allowing patients to focus their own attention on their environment, relatives and friends and then explaining an improvement of HRQoL. On the other hand, precuneus has been also involved in the theory of mind (ToM) [50,51] which is conceived as a set of abilities that enable humans to understand other peoples' mental states and intentions. In particular, ToM is interrelated with self-focus attention and self-emotional awareness [51]. Moreover, previous studies have reported that ToM performance was associated with an increased perfusion of the precuneus [28,31]. Interestingly, Wolkenstein et al. [52] found that depressed patients were more accurate in decoding negative than neutral and positive mental states. The precuneus hypoperfusion found in our study may reflect a better attention for positive episodes and a decreased emotional response to negative thoughts [43] and thus be associated with improved mental health HRQoL concerning feelings of nervousness, peacefulness, and happiness [28,31].

Our findings should be considered in treatment of patients with MDD, supporting the use of therapeutic interventions targeting self-focus and SRP, such as mindfulness-based cognitive therapy [53] or cognitive base-therapy, to improve HRQoL. On the other hand, the impact of rTMS applied specifically to precuneus in patients with MDD on HRQoL should be investigated in future studies.

Lastly, the links between precuneus perfusion and HRQoL on the one hand, and those between HRQoL and depression on the other hand, help to better understand the mechanism of action of rTMS. The impact of rTMS may not only relieve the symptoms of MDD, but also affects other relevant psychosocial domains measured by HRQoL, which may in turn influence mood improvement. In accordance with this hypothesis, recent studies have shown HRQoL to be a prognostic factor associated with clinical outcome in various chronic diseases [54,55]. These findings may provide a support to integrate HRQoL in clinical practice, as complementary information to that traditionally collected in psychiatry.

### Limitations

Several limitations have to be considered in this exploratory study. The first limitation is the small sample size (n = 15); the lack of power may explain the contrast between the non-significant statistical results and the small to moderate level of effect sizes. Second, the absence of control group limits our ability to attribute the HRQoL changes exclusively to rTMS. However, although controlled trials are appropriate for establishing potentially causal associations, more naturalistic studies have the advantage of assessing “real life” situations of patients under treatment. These distinct limitations, also present in the previous reports [8,25], should be considered in future studies. Third, we used the standard 5-cm localization procedure for applying rTMS over the right DLPFC, instead of using a neuronavigational method, which seems to enhance response to rTMS [56]. Fourth, the unbalanced sample regarding gender might have bias our findings. However, the influence of gender on HRQoL is still unclear [57-59], and gender is not associated with difference in rTMS outcomes [60]. Sixth, some patients were receiving medical treatment during the study, including benzodiazepins that modify cortical excitability. Therefore, the effects of these medications on HRQoL and rCBF cannot be excluded. Finally, the use a self-rating subjective scale (BDI-II) to assess the severity of depression could be criticized. However, studies support a satisfactory convergent validity between BDI-II and Hamilton Depression Rating Scale or Clinical Global Impression [61,62].

## Conclusion

This study suggests low-frequency rTMS can improve HRQoL, through its role-physical problems dimension, in patients with MDD. This improvement is associated with a decreased perfusion of the precuneus, a brain area involved in self-focus and self-processing, arguing for a neural substrate to the impact of rTMS on HRQoL.

## Abbreviations

HRQoL: Health-related quality of life; MDD: Major depressive disorder; DSM-IV: Diagnostic and statistical manual of mental disorder, fourth edition; rTMS: Repetitive transcranial magnetic stimulation; DLPFC: Dorsolateral prefrontal cortex; SPECT: Single-photon emission computed tomography; rCBF: Regional cerebral blood flow; SPM: Statistical parametric mapping; SD: Standard derivation; PF: Physical functioning; RPP: Role—Physical Problems; VIT: Vitality; BP: Bodily pain; MH: Mental health; REP: Role—emotional problems; SF: Social functioning; GH: General health; MCS: Mental composite score; PCS: Physical composite score; BDI-II: Beck depression inventory; SRP: Self-referential processing; ToM: Theory of Mind.

## Competing interests

The authors have declared that there are no conflicts of interest in relation to the subject of this study.

## Authors’ contributions

RD, RR, EG, PA and LB wrote the manuscript. All authors designed the study and wrote the protocol. RD, RR, EG, CL and LB managed the literature searches and analyses. EG and LB managed the statistical analysis. All authors contributed to and approved the final manuscript.
